# A rare case of Seymour fracture in an adult with non-fused growth plates

**DOI:** 10.1080/23320885.2021.1927738

**Published:** 2021-05-19

**Authors:** Mohammed Farid, Mohamed Shibu

**Affiliations:** a Plastic Surgery, Queen Elizabeth Hospital, Birmingham, UK; b Plastic Surgery, The Royal London Hospital, London, UK

**Keywords:** Seymour fracture, growth plates, adult, non-fusion

## Abstract

The mechanism for growth plate fusion is not fully understood yet. We present the first reported Seymour fracture (Salter Harris I) in an adult with failed growth plate fusion. The management of Seymour fractures should be according to radiological bone age rather than actual age.

## Introduction

Seymour fracture was first described in 1966 as a transverse extra-articular open fracture of the distal phalanx associated with nail bed injury in children [1]. It’s a type of Salter-Harris I/II with a transverse fracture through the growth plate and includes juxta-epiphyseal fractures [2]. It’s so-called Seymour-type fracture by adults when localised at the same location as in children with Seymour’s fracture, but not related to growth plate [3]. The management of Seymour fracture is either operative or non-operative as described in the literature [4]. A successful strategy is to ensure appropriate management of fracture healing without damaging growth plates which may lead to early bony fusion. Nonetheless, the detailed mechanism for growth plate fusion is not fully understood. Bone growth is influenced by a number of nutritional, cellular, hormonal and genetic factors [5]. One hypothesis indicates that delayed growth plate fusion may be related to the deficiency or resistance to oestrogen in adolescence. We present a rare case of single digit (non-dominant) Seymour fracture in an adult with no-fusion of growth plates in all digits. This was managed with surgical exploration, fracture reduction, axial K-wire fixation and nail bed repair.

## Case report

**Figure 1.**
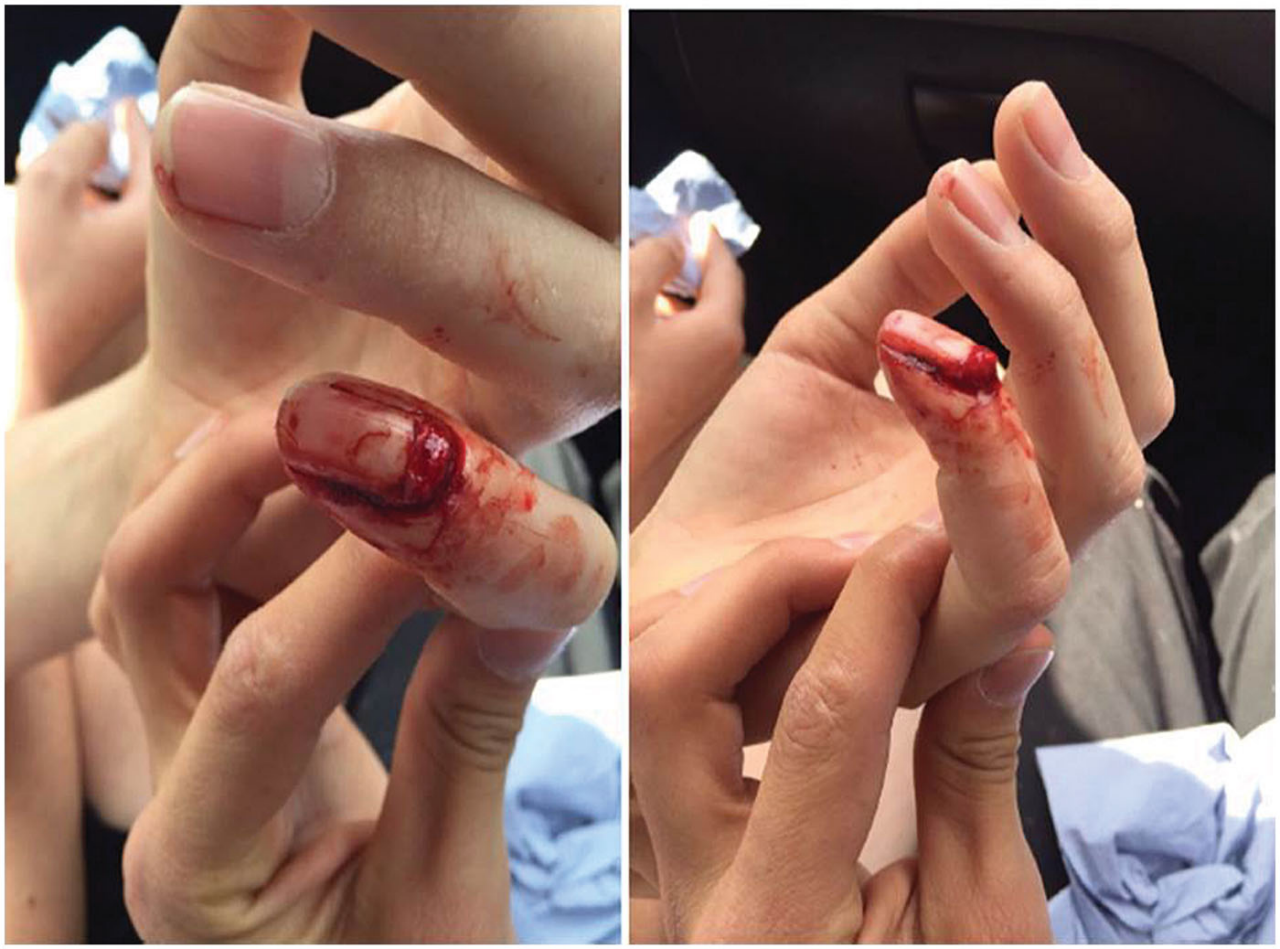
Left Little Finger Nail plate Avulsion and Loss of DIPJ Extension.

A 19 year old Caucasian man presented with a left little finger mallet injury open juxta – epiphyseal fracture of distal phalanx (Figure 1). This was consisted with a Seymour type fracture (Salter Harris I) with involvement of nail bed, germinal matrix and ungual subluxation. The mechanism was due to a fall from a bicycle 2 days prior to clinic visit. Five days post injury, this was managed with open reduction, nail bed repair (6/0 vicryl rapide) and one axial K-wire (0.9 mm) fixation. Further clinical assessment based on Greulich and Pyle (GP) and Tanner-Whitehouse (TW) revealed bone age of 14.5 and 14.3 years respectively. The K-wire was removed 4 weeks post-operatively and DIPJ was splinted for further 2 weeks. The patient regained full extension of DIPJ post rehabilitation was followed–up for 6 months without any functional deficit or nail deformity. A referral to endocrinology team was made to rule out any growth hormone, thyroid or pituitary related diagnoses.

**Figure 2.**
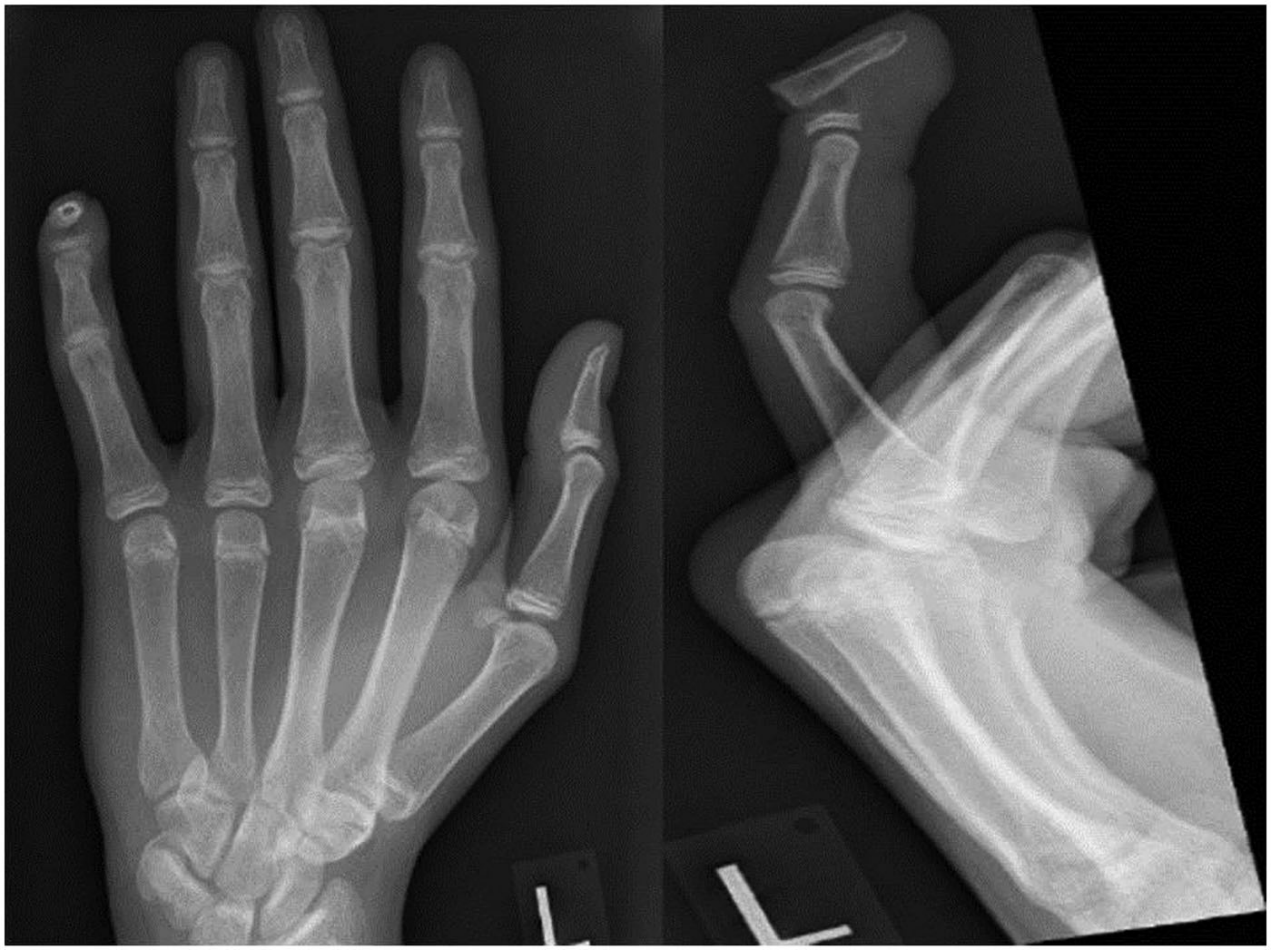
Left Little Finger AP + Lateral Radiographs – Seymour Fracture.

**Figure 3.**
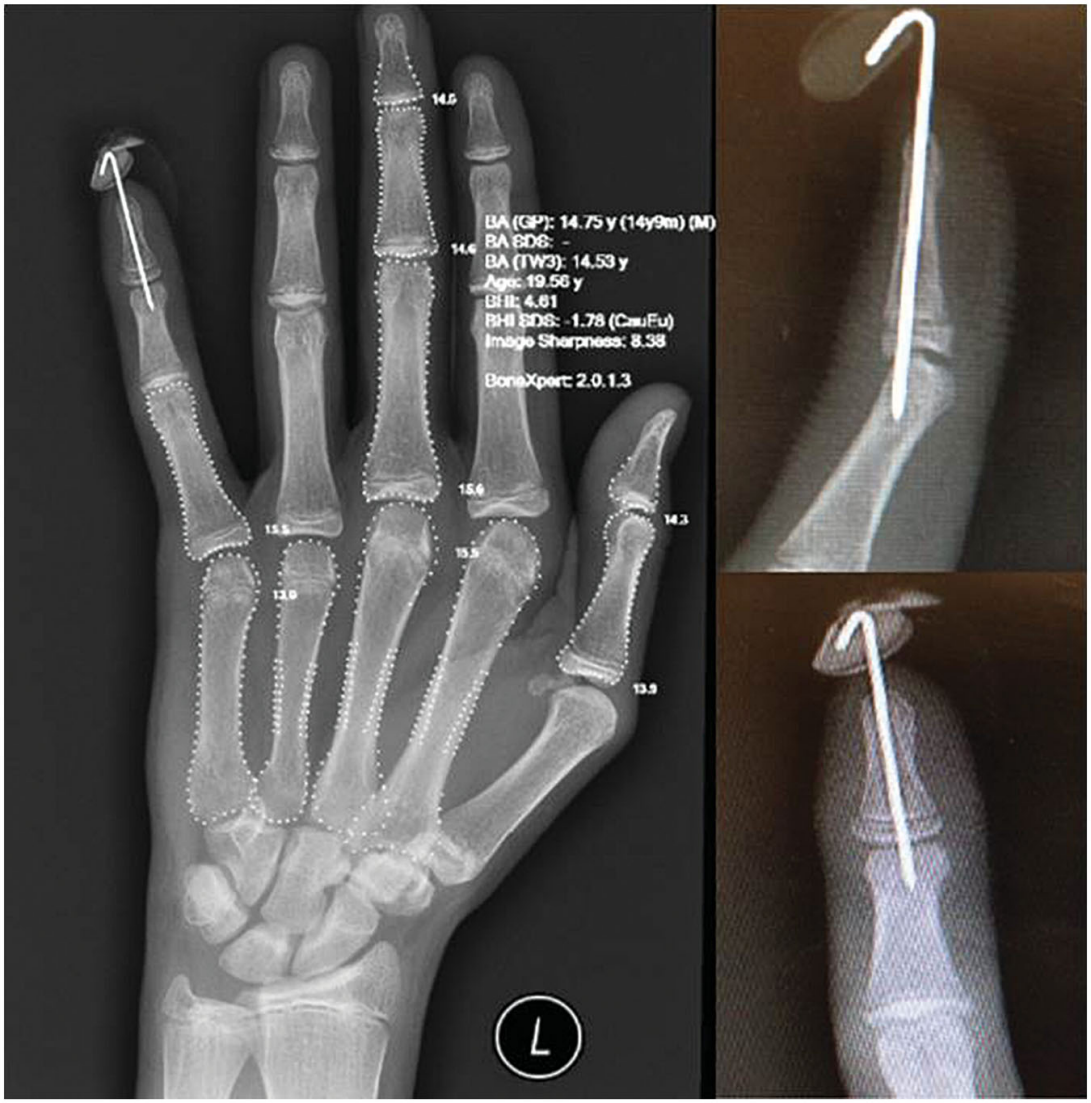
Post-operative images with Axial K-wire post reduction (AP + Lateral) demonstrating bone age.

The line of the Seymour fracture localised 1–2 mm distal to the insertion of the extensor tendon and clinically may resemble a mallet like finger due to the insertion points of the flexor digitorum profundus proximal to the fracture line causing a deformity where the shaft of the distal phalanx is flexed and the epiphysis remains extended [1,3]. The initial radiographs (Figure 2) demonstrated open juxta-epiphyseal Seymour fracture of the distal phalanx and non-fused growth plate in all digits and joints of left hand. Broad spectrum antibiotics were indicated to reduce the risk of infection. Our approach was surgical exploration of fracture site, anatomical alignment of bone and 0.9 mm K-wire stabilisation (Figure 3). The nail bed was repaired with (6/0 vicryl rapide). The axial K-wire was removed 4 weeks post-operatively and the distal interphalangeal joint (DIPJ) was splinted for an additional 2 weeks. Gradual range of movement exercises was commenced by our hand therapists after the removal of the splint. The patient regained full extension of the DIPJ post rehabilitation without any functional deficit or nail deformity at 6 months follow-up.

In order to confirm the bone age, further radiological assessment was based on Greulich and Pyle (GP) and Tanner-Whitehouse (TW) criteria [6] which revealed 14.75 and 14.3 years, respectively. This finding suggests bone age being five years less than the actual age of the patient.

## Discussion

The underlying reason for this discrepancy between actual and bone age was better determined by growth plate anatomy and factors affecting fusion. Growth plate in children has three zones (resting, proliferative, hypertrophic) located between epiphysis and metaphysis. Each zone has chondrocytes differentiation at a variable pace particularly during pubertal growth spurt. Other processes including extracellular matrix secretion, osteoblast differentiation and vascular invasion also occur. The exact mechanism of epiphyseal fusion and growth plate arrest at the end of puberty is still not completely understood. The delay in fusion is noted in those with oestrogen deficiency and precocious puberty [5]. Male patients with oestrogen resistance and aromatase deficiency showed incomplete epiphyseal closure. It highlights that oestrogen plays a role in stimulating growth plate maturation and epiphyseal fusion *via* a poorly understood mechanism. One of the important cellular factors is the differentiation and ageing of chondrocytes in the growth plate. Various paracrine factors play a role in chondrocyte differentiation, vascularisation and osteoblast ossification [7]. Various medical reasons may contribute to the delay in the fusion of growth plates till adulthood. We highlight the need to engage in a well-informed discussion about this diagnosis with the patient and refer to endocrinology team for further investigations accordingly.

In this case, the management of Seymour fractures was operative to remove interposed tissue in the fracture site. Interposition of germinal matrix into fracture site may result in nail plate deformity, physeal arrest and osteomyelitis if untreated promptly [2,8]. The recurrent interposition of tissue in the fracture site is prevented by repairing the soft tissue. The subsequent options to stabilise the fracture is either splinting only or a temporary K-wire [4]. We opted for the latter option to ensure no subsequent fracture displacement while healing is achieved.

The assessment of bone age was based on GP and TW methods radiologically. While, the actual age ideally correlates with bone age, but was not a match in this rare case. We conclude that the management of Seymour fractures should be guided by bone age, clinical, radiological and surgical findings rather than actual age.
